# Divergent effects of urban particulate air pollution on allergic airway responses in experimental asthma: a comparison of field exposure studies

**DOI:** 10.1186/1476-069X-11-45

**Published:** 2012-07-06

**Authors:** James G Wagner, Masako Morishita, Gerald J Keeler, Jack R Harkema

**Affiliations:** 1 Department of Pathobiology and Diagnostic Investigation, College of Veterinary Medicine, Michigan State University, East Lansing, MI 48824, USA; 2 Department of Environmental Health Sciences, University of Michigan, 1415 Washington Heights, Ann Arbor, MI 48109, USA

**Keywords:** Particulate matter, Asthma, Pulmonary inflammation, Air pollution, Concentrated air particles

## Abstract

**Background:**

Increases in ambient particulate matter of aerodynamic diameter of 2.5 μm (PM_2.5_) are associated with asthma morbidity and mortality. The overall objective of this study was to test the hypothesis that PM_2.5_ derived from two distinct urban U.S. communities would induce variable responses to aggravate airway symptoms during experimental asthma.

**Methods:**

We used a mobile laboratory to conduct community-based inhalation exposures to laboratory rats with ovalbumin-induced allergic airways disease. In Grand Rapids exposures were conducted within 60 m of a major roadway, whereas the Detroit was located in an industrial area more than 400 m from roadways. Immediately after nasal allergen challenge, Brown Norway rats were exposed by whole body inhalation to either concentrated air particles (CAPs) or filtered air for 8 h (7:00 AM - 3:00 PM). Both ambient and concentrated PM_2.5_ was assessed for mass, size fractionation, and major component analyses, and trace element content. Sixteen hours after exposures, bronchoalveolar lavage fluid (BALF) and lung lobes were collected and evaluated for airway inflammatory and mucus responses.

**Results:**

Similar CAPs mass concentrations were generated in Detroit (542 μg/m^3^) and Grand Rapids (519 μg/m^3^). Exposure to CAPs at either site had no effects in lungs of non-allergic rats. In contrast, asthmatic rats had 200% increases in airway mucus and had more BALF neutrophils (250% increase), eosinophils (90%), and total protein (300%) compared to controls. Exposure to Detroit CAPs enhanced all allergic inflammatory endpoints by 30-100%, whereas inhalation of Grand Rapids CAPs suppressed all allergic responses by 50%. Detroit CAPs were characterized by high sulfate, smaller sized particles and were derived from local combustion sources. Conversely Grand Rapids CAPs were derived primarily from motor vehicle sources.

**Conclusions:**

Despite inhalation exposure to the same mass concentration of urban PM_2.5_, disparate health effects can be elicited in the airways of sensitive populations such as asthmatics. Modulation of airway inflammatory and immune responses is therefore dependent on specific chemical components and size distributions of urban PM_2.5_. Our results suggest that air quality standards based on particle speciation and sources may be more relevant than particle mass to protect human health from PM exposure.

## Background

Epidemiological studies have implicated several different pollutants in urban air mixtures as contributing to asthma morbidity and mortality. These range from coarse, fine and ultrafine particulate matter (PM) [1,2], gases such as carbon monoxide, nitrogen dioxide, sulfur dioxide and ozone [3-5], and specific particulate species such organic and inorganic carbons, sulfate, nitrates and metals [6-8]. Clearly, some of these components may simply be markers for the causative agent(s), but each likely contributes to the respiratory effects in each community and unique exposure scenario.

Fine PM (< 2.5 μm; PM_2.5_) is frequently cited as a key factor in hospital admissions for asthma [9,10], and severe asthma attacks and declining lung function, especially in children [11,12]. Although plausible mechanisms for airway exacerbation by PM_2.5_ have been proposed (e.g., oxidative stress, NFkB, activation, etc.), the mode of action for PM_2.5_ to exacerbate allergic airways responses has not been clearly defined. Airborne particles can exist in many physicochemical forms and as complex mixtures of acids, metals, and organic and inorganic carbon compounds [13], and as such provides PM_2.5_ with a range of potential bioactive properties that can vary with seasons and location.

Over the last decade we have used a mobile laboratory with particle concentrators to conduct real-time, community-based inhalation exposures of laboratory animals to concentrated air particles (CAPs) derived from ambient PM_2.5_ with the goal of determining the relationships of PM components to health outcomes [14,15]. For example we have linked exacerbation of allergic mucus and inflammatory responses in rats after multiple exposures to Detroit CAPs to local combustion sources and specific metals such as La, Ni, S and Mn [15,16]. In similar studies in Grand Rapids, MI where there are fewer industrial emission sources compared to Detroit, CAPs was derived mostly of organic carbon, and multiple days of exposure induced relatively modest inflammatory responses in allergic rats [17]. However these latter exposures were also marked by high variability in CAPs concentrations and extreme elevations in sulfate concentrations, so it is difficult to determine what component of PM_2.5_ are most responsible for biological effects in allergic airways.

In the present study we used a rodent model of experimental asthma to extend an earlier comparative evaluation of Detroit and Grand Rapids CAPs where a single 8 h exposure period resulted in similar particle concentrations [18]. Independent of PM mass, we were able to test the hypothesis that PM_2.5_ from both Michigan cities would enhance airway inflammation and mucous responses, and that the effect of PM from Detroit would be more pronounced than that from Grand Rapids. As such, we were able to focus on the distinct qualitative differences in CAPs content and avoid uncertainties of multiple exposures to variable PM concentrations and components. Here we describe divergent responses on allergic inflammation and airway mucus responses by single exposure to Detroit and Grand Rapids CAPs that can only be explained by the qualitative differences in chemical and physical characteristics. Because of the similar PM mass concentrations in each discrete exposure, we can make clear comparisons of PM attributes to their associated effects on allergic airways disease.

## Methods

### Site descriptions, Detroit and Grand Rapids, MI

**Figure 1:**
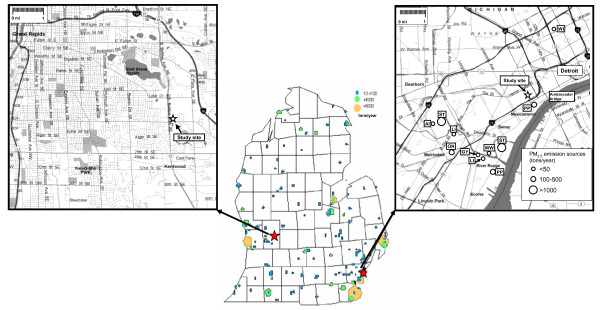
**Exposure study sites. **Maps of Grand Rapids (left) and southwest Detroit, MI (right) showing the locations of the study sites and major industrial sources for PM_2.5_ (USEPA-Air Data NET, 1999) (AI: auto industries, CE: cement industries, GY: gypsum industries, LI: lime industries, OR: oil refineries, PP: coal-fired power plants, ST: iron/steel industries, WI: waste incinerator, WW: waste water treatment and sludge incinerator).

Separate 8-h exposure studies were conducted in Detroit and Grand Rapids, MI on 29 July 2002 and 11 August 2003, respectively. Major point emission sources for PM_2*.*5_ in Michigan are indicated in Figure 1[19]. Emission sources in southwest Detroit that likely impact the local area where we conduct inhalation studies at Maybury Elementary School have been previously described [20]. This area of the city has heavy industries, including iron-steel manufacturing, coke ovens, chemical plants, refineries, sewage sludge incineration, and coal-fired utilities [21]. In addition, southwest Detroit experiences heavy motor vehicle traffic, both passenger car and diesel truck traffic, due to its proximity to major interstates international traffic with Canada at the Ambassador Bridge.

Relative to Detroit, western Michigan frequently experiences elevated levels of transported secondary air pollutants that are generated from precursor emission sources in Illinois, Indiana, Ohio, Wisconsin, and Canada. These pollutants are transported and chemically react as they move across Lake Michigan or move from Ohio River Valley into Michigan. Our second sampling location was in Grand Rapids, Michigan’s second largest city, and located within 60 m of a freeway.

### Inhalation exposure studies

#### *Mobile air research laboratory*

AirCARE 1 was designed and constructed collaboratively by Michigan State University (MSU) and the University of Michigan to conduct air pollution health effects studies [14]. The inhalation exposure lab contains a Harvard-type fine particle concentrator and two reinforced stainless steel Hinners-type whole-body inhalation chambers with volume of 0.32 m^3^, and hold a single level of 16 rats. One chamber was connected to the concentrator for exposure to concentrated air particles (CAPs), while the second chamber was supplied with HEPA-filtered clean air at the same flow rate as the other chamber. The fine particle concentrator is a 3-stage aerosol concentrator that utilizes virtual impactors to increase the concentration of particles (size range 0.1–2.5 μm) by an approximate factor of 30 [22].

#### *Animals*

Male Brown Norway rats (Charles River, Portage, MI), 10–12 weeks of age, were assigned to one of four experimental groups (*n* = 8/group) at each exposure site. Animals were free of pathogens and respiratory disease, and used in accordance with guidelines set forth by the Institutional Animal Care and Use Committee at MSU. Animals were initially housed in MSU animal facilities before being transferred on site to AirCARE1. Specific exposure protocols have been described in detail [14]. In AirCARE1, the rats were housed individually in rack-mounted stainless steel wire cages in Hazleton chambers (HC-1000, Lab Products, Maywood, NJ), and then transferred to Hinners exposure chambers during CAPs inhalation exposures.

#### *Experimental asthma protocols*

**Figure 2:**
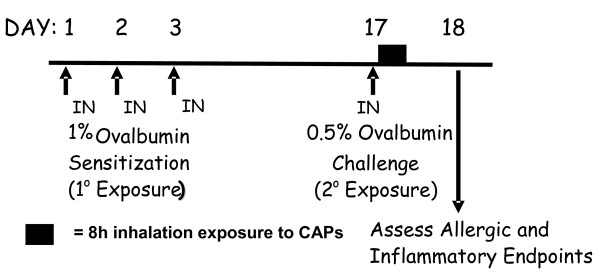
**Experimental protocol of CAPs exposure during allergen challenge. **Brown Norway rats were sensitized with intranasal (IN) ovalbumin (OVA) for 3 consecutive days, and then challenged with IN OVA or vehicle 14 days later. Within 30 min rats were exposed to filtered air or CAPs for 8 h, and allergic airway responses were assessed one day later.

Rats were sensitized to chicken albumin (ovalbumin; OVA; Sigma Chemical Co.) by intranasal (IN) instillation of OVA (0% or 0.5% in saline, 150 *μ*l/naris) for 3 consecutive days (Figure 2). Rats were instilled IN while under light anesthesia (4% halothane in oxygen). Fourteen days later rats were challenged with IN saline or OVA (i.e., asthmatic rats). This airway sensitization and challenge protocol produces allergic airway disease which we have characterized with pathological endpoints of secretory cell metaplasia, mucus hypersecretion and inflammatory cell recruitment 24 h after allergen challenge [23]. OVA sensitization and challenge treatment occurred in AirCARE1. Approximately 30–40 min after a single intranasal administration of OVA allergen, rats were placed into Hinners exposure chambers and exposed to either CAPs or filtered air for 8 h, from 7:00 AM–3:00 PM. After exposure, animals were transported back to laboratories at Michigan State University.

#### *Tissue collection and analysis*

Sixteen hours after the end of inhalation exposure (approximately 9:00 AM the following morning), rats were anesthetized with sodium pentobarbital and euthanized by exsanguination. The trachea was cannulated, and the heart and lung removed *en bloc*. The bronchus to the left lung was temporarily closed with a hemostatic clamp, and 5 ml of sterile saline was instilled through the tracheal cannula and withdrawn to recover bronchoalveolar lavage fluid (BALF) from the right lung lobes. A second saline lavage was performed and combined with the first.

After lavage, the right lung lobes were ligated and removed for future analyses. The clamp was removed from the left bronchus, and the left lobe was inflated under constant pressure (30 cm H_2_O) with neutral-buffered formalin for 2 h, while immersed in a large volume of fixative. Twenty-four hours later, two sections were excised from the left lung lobe at the level of the 5th and 11th airway generation, to sample proximal and distal airways, respectively. Tissue blocks were then embedded in paraffin, and 5–6 μm thick sections were cut from the anterior surfaces. Lung sections were stained with hematoxylin and eosin (H&E) for routine histology and with Alcian Blue (pH 2.5)/Periodic Acid-Schiff to detect intraepithelial mucosubstances (IM).

Total leukocytes in BALF were enumerated with a hemocytometer, and fractions of eosinophils, neutrophils, macrophages, and lymphocytes were determined in a cytospin sample stained with Diff-Quick. Total protein was determined by the bicinchoninic acid method. Secretion of mucosubstances into airways was estimated by an ELISA for mucin glycoprotein 5 AC using a mouse monoclonal antibody to the human MUC5AC protein (Neomarkers, Fremont, CA) that has reactivity to the rat rMuc5AC core protein. Bound primary antibody was detected with a biotinylated secondary antibody and quantitated using horseradish-peroxidase-conjugated avidin/biotin complex (ABC Reagent; Vector Laboratories, Burlingame, CA) and a fluorescent substrate (QuantaBlue; Pierce Chemical, Rockford, IL).

To estimate the amount of the intraepithelial mucous (IM) in the respiratory epithelium lining the axial pulmonary airways from proximal and distal lung sections, the volume density (Vs) of AB/PAS-stained mucosubstances was quantified using computerized image analysis and standard morphometric techniques. Briefly, the area of AB/PAS-stained mucosubstance was estimated by setting colorized thresholds to highlight only IM, and these areas were then automatically calculated using the public domain NIH Image program (http://rsb.info.nih.gov/nih-image/). The length of the basal lamina underlying the surface epithelium was calculated from the contour length of the basal lamina. The volume of stored mucosubstances per unit of surface area of epithelial basal lamina was estimated using the method described in detail as elsewhere [24], and is expressed as nanoliters of intraepithelial IM per mm^2^ of basal lamina (i.e., volume density).

### Ambient gaseous pollutant measurements

Ozone (O_3_), sulfur dioxide (SO_2_), nitrogen oxides (NOx) and carbon monoxide (CO) were measured continuously. Meteorological parameters including temperature, relative humidity, precipitation, wind speed and direction were monitored continuously atop a 10-m tower attached to AirCARE1.

### CAPs collection and characterization

Sample handling, processing, and analysis took place in a Class 100 ultra-clean laboratory at the University of Michigan Air Quality Laboratory, designed for ultra-trace element analysis with an emphasis on low-level environmental determinations. Collection and analysis of CAPs samples from the Harvard Fine particle concentrator in AirCARE1 have been described in detail elsewhere [14,15]. The output flow from the third stage of the concentrator is nominally 50 LPM, with 15 LPM of the flow used for sample collection and the remaining 35 LPM delivered to the animal exposure chambers. CAP mass was determined on 47-mm Teflon filters (PTFE, Gelman, Ann Arbor, MI) collected at flow rates of 3 LPM. Annular denuder/filter packs were used to collect the acidic gaseous species and inorganic ions including sulfate, nitrate and ammonium as described previously [25]. Size-fractionated particle sampling was performed using a six-stage micro-orifice impactors (MOIs). Sample volumes of air were determined using a calibrated DTM (Schlumberger, Owenton, KY), and a calibrated rotameter (Matheson Inc., Montgomeryville, PA) was used to check flow rates at the beginning and end of each sampling period. A TEOM 1400a (Rupprecht and Patashnick Inc., Albany, NY) was placed in line to continuously monitor the CAPs concentrations.

For all chemical and physical analyses in the laboratory, field blanks, filter-lot blanks, replicate analyses, and externally certified standards were incorporated for quality assurance and quality control purposes. These analyses are briefly described here.

#### *Gravimetric analysis*

Gravimetric analysis was performed using a microbalance (MT-5 Mettler Toledo, Columbus, OH) in a temperature/humidity-controlled clean laboratory as described in Federal Reference Method [26].

#### *Organic and elemental carbon*

CAP samples collected on quartz filters were stored at temperatures at −40°C after sampling and analyzed for carbonaceous aerosols by a thermal-optical analyzer using NIOSH Method 5040 (Sunset Labs, Forest Grove, OR).

#### *Major ions and acid aerosol*

Denuders, Teflon filters and carbonate-coated backup filters were extracted in Milli-Q ultrapure water. Extracts were then analyzed for anions and cations for gaseous species and major ions by chromatography (Model DX-600, DIONEX, Sunnyvale, CA).

#### *Trace element analysis*

After completion of gravimetric analysis, Teflon sample filters were placed in 15 mL acid-cleaned centrifuge tubes and were wetted with 150 μl of ethanol before extraction in 10 ml of 10% HNO_3_. The extraction solution was then sonicated for 48-h in an ultrasonic bath, and then allowed to passively acid-digest for a minimum of 2 weeks. Sample extracts were then analyzed for a suite of trace elements using high-resolution inductively coupled plasma-mass spectrometry

(ELEMENT2, Thermo Finnigan, San Jose, CA).

### Statistical analysis

Data describing the pulmonary responses in BN rats is expressed as mean ± standard error of the mean. Differences in treatment and exposure related effects of OVA and CAP were determined with ANOVA and individual comparison by Student Newman-Kuels *post hoc* test, with criterion for significance set at *p* ≤ 0.05 (SigmaStat 11.0; Jandel Scientific). Statistical analyses including correlation, multiple regression and analysis of variance for all of the gaseous pollutants and PM components were performed using a statistical analysis system (SAS 9.1, SAS Institute, Inc., Cary, NC). Data describing the parameters of particle number, mass, and components were expressed as the mean value ± the standard deviation. Statistical significance was tested using Tukey–Kramer *post hoc* test. The criterion for statistical significance was p ≤ 0.05.

## Results

### Bronchoalveolar lavage

**Figure 3:**
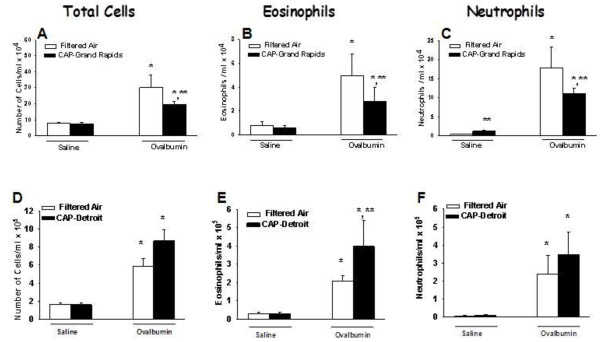
**Effect of CAPs exposure on BALF cellularity. **Animals were sensitized and challenged with saline (white bars) or with OVA (black bars) immediately prior to exposure to CAPs in Grand Rapids **(A, B, C)**, or Detroit **(D, E, F)**. Twenty four hours after the CAPs exposure bronchoalveolar lavage fluid was collected and total cells, eosinophils, and neutrophils were enumerated as described in Methods. * = significantly different from respective group challenged with saline; ** = significantly different from respective group exposed to filtered air; n = 8/group; *p* < 0.05.

Sensitization and challenge of rats to OVA caused an increase in BAL fluid of total cells, eosinophils and neutrophils compared to Air/Saline rats in both Detroit and Grand Rapids studies (Figure 3). All these allergic inflammatory responses were attenuated by approximately 50% by exposure to Grand Rapids CAPs (Figure 3A, B, C). By comparison, BALF eosinophils were increased 100% by Detroit CAPs exposure (Figure 3D, E, F), with a clear trend for increases in both total cells and neutrophils. BAL content of macrophages and lymphocytes were not altered by OVA challenge or CAPs exposure at either site.

**Figure 4:**
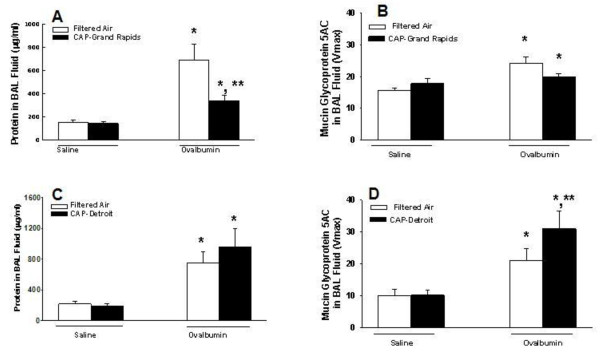
**Effect of CAPs exposure on mucus hypersecretion and BALF protein. **Animals were sensitized and challenged with saline (white bars) or with OVA (black bars) immediately prior to exposure to CAPs in Grand Rapids (**A, B**), or Detroit (**C, D**). Twenty four hours after the CAPs exposure bronchoalveolar lavage fluid was collected and content of total protein and mucin glycoprotein 5 AC were determined as described in Methods. * = significantly different from respective group challenged with saline; ** = significantly different from respective group exposed to filtered air; n = 8/group; *p* < 0.05.

Airway mucus secretion was increased by OVA sensitization and challenge as indicated by an increase in immunoreactive Muc5AC in BAL fluid compared to control rats in both Detroit and Grand Rapids studies (Figure 4B, D, respectively). In asthmatic rats exposed to Grand Rapids CAPs, mucus secretion was diminished to control levels, whereas BALF mucus was increased by 50% by Detroit CAPs in asthmatic rats. Protein content in BAL fluid was also increased by OVA challenge during both studies (Figure 4A, C). Exposure to Grand Rapids CAPs inhibited OVA-induced protein increases by 84% (Figure 4A). Modest increases in BALF protein collected from asthmatic rats by Detroit CAPs were not significant (Fig 4B).

### Histopathology

In Grand Rapids and Detroit, Air/Saline and CAPs/Saline rats had no exposure related histopathology in pulmonary tissue sections examined by light microscopy. The principal morphologic lesions in the lungs of asthmatic rats were an allergic bronchiolitis and alveolitis (allergic bronchopneumonia). OVA-induced inflammatory and epithelial lesions in the conducting airways involved the large diameter, proximal axial airways and the small diameter, distal preterminal and terminal airways. Inflammatory and epithelial lesions were usually more severe in the more proximal axial bronchioles compared to those in the more distal preterminal and terminal bronchioles. OVA-induced bronchiolitis was characterized by peribronchiolar edema associated with a mixed inflammatory cell influx of eosinophils, lymphocytes, plasma cells, and occasional neutrophils. Peribronchiolar inflammation was principally located in the subepithelial interstitial tissues (e.g., lamina propria and submucosa) with markedly fewer inflammatory cells in the surface epithelium lining these airways.

**Figure 5:**
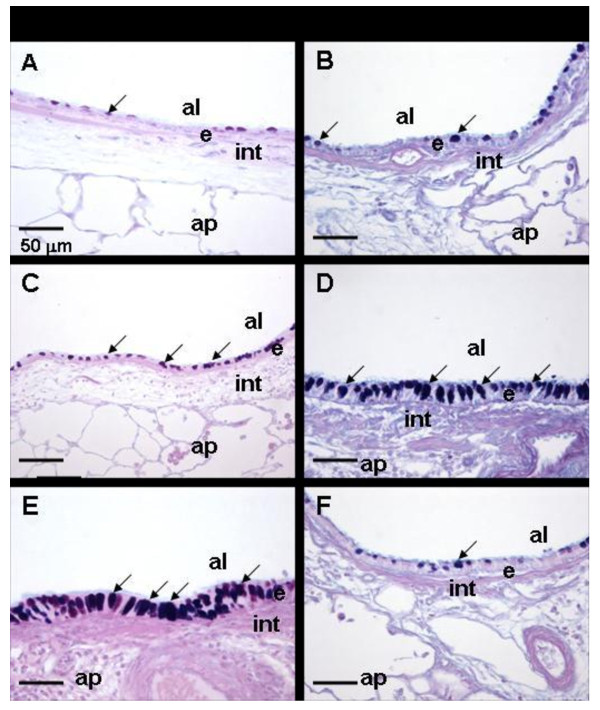
**Effect of CAPs exposure on mucous cell metaplasia in allergic pulmonary airways. **Photomicrographs of the respiratory airway epithelium lining the axial airway in lungs of rats sensitized and challenged with saline (**A, B**), or with Ovalbumin (**C-F**). Rats were exposed to filtered Air (**A-D**) or to CAPs in Detroit (**E**) or Grand Rapids (**F**). Tissues were stained with AB/PAS to identify intraepithelial mucosubstances (identified by arrows). al = airway lumen; ap = alveolar parenchyma; e = epithelium; int = interstitium; arrows = intraepithelial mucosubstances. Magnification bar = 50 μm.

Asthmatic rats exposed to filtered air had a mucous cell metaplasia/hyperplasia (MCM) with increased amounts of AB/PAS-stained mucosubstances in the surface epithelium (i.e., intraepithelial mucosubstances; IM) lining the affected large diameter bronchioles, including the proximal and distal axial airways (Figure 5 C, D). Saline-instilled and filtered air- or CAPs-exposed rats had significantly fewer mucous cells and IM compared to the asthmatic rats. There was no significant difference in the amounts of IM between saline-instilled rats exposed to CAPs and saline-instilled rats exposed to only filtered air.

In addition to the perivascular and peribronchiolar lesions, there were varying sized focal areas of allergic alveolitis in the lung parenchyma. These alveolar lesions were characterized by accumulations of large numbers of alveolar macrophages, epithelioid cells, and eosinophils, with lesser numbers of lymphocytes, monocytes and plasma cells, in the alveolar airspace. Often the alveolar septa in these areas of alveolitis were thickened due to type II pneumocyte hyperplasia and hypertrophy, intracapillary accumulation of inflammatory cells, and capillary congestion.

Asthmatic rats exposed to Detroit PM_2.5_ developed more severe allergic bronchopneumonia than asthmatic rats exposed to filtered air. This was reflected both in the severity and distribution of the allergic bronchiliolitis and alveolitis.

CAP/OVA exposed rats also had more MCM in the epithelium lining the large diameter axial airways compared to OVA-instilled rats exposed only to filtered air (Figure 5C, E).

Asthmatic rats exposed to Grand Rapids CAPs had more modest attenuation of bronchopneumonia than asthmatic rats exposed to filtered air. This was reflected in the distribution of severity of the bronchiolitis and alveolitis. CAPs-exposed asthmatic rats also had decreased MCM in the epithelium lining the large diameter axial airways compared to filtered air-exposed asthmatic rats (Figure 5 D, F).

### Morphometry of intraepithelial mucosubstances (IM)

**Figure 6:**
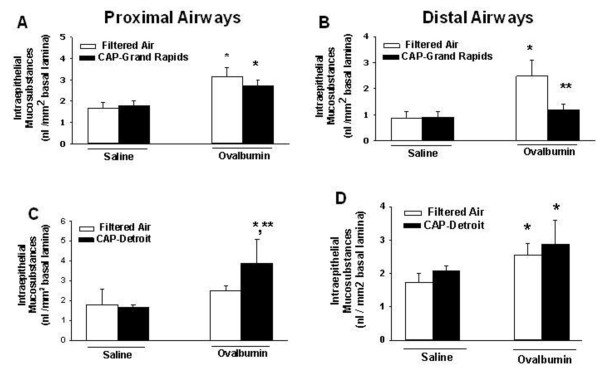
**Effect of CAPs exposure on intraepithelial mucosubstances (IM). **Animals were sensitized and challenged with saline (white bars) or with OVA (black bars) immediately prior to exposure to CAPs in Grand Rapids (**A, B**), or Detroit (**C, D**). Twenty four hours after the CAPs exposure lungs were processed and stained with AB/PAS and intraepithelial mucus was quantified in proximal (**A, C**) and distal (**B, D**) conducting axial airways as described in Methods. * = significantly different from respective group challenged with saline; ** = significantly different from respective group exposed to filtered air; n = 8/group; *p* < 0.05.

Exposure to PM_2.5_ in either Detroit or Grand Rapids did not significantly affect IM in airways of rats sensitized and challenged with saline (i.e., non-allergic rats) (Figure 6). However OVA sensitization and challenge induced mucous cell metaplasia in proximal and distal airways of the rats used in Grand Rapids, and in distal airways in Detroit rats. Exposure to Grand Rapids PM_2.5_ blocked IM increases in asthmatic rats to control levels in distal airways (Figure 6B). Conversely, Detroit PM_2.5_ caused a 75% increase IM in proximal airways of asthmatic rats (Figure 6C). PM_2.5_ -induced changes in IM of asthmatic rats were not significant in other airways.

### Physical and chemical characterization of CAPs

#### *CAPs mass concentration*

Concentrator performance was continually monitored via TEOM readings during exposures to ensure that CAPs reflected the variation in ambient PM_2*.*5_; the evaluation of the concentrator performance is described in detail elsewhere [14,27]. The ambient PM_2*.*5_ concentrations during the exposures were 18.3 and 16.1 μg/m^3^ in Detroit and Grand Rapids, respectively. The average CAPs concentrations measured during the 8-h exposure periods in Detroit and Grand Rapids were 542 μg/m^3^ and 519 μg/m^3^, respectively. As such, Concentration Enrichment Factors (CEF) for Detroit and Grand Rapids were 29.6 (542/18.3) and 32.2 (519/16.1), respectively, or very close to the expected performance of 30 CEF.

#### *CAPs size distribution*

Table 1 provides a comparison of mass size distributions of CAPs in six discrete size fractions (<0.18 μm, 0.18–0.6, 0.6–1, 1–2.5, 2.5–5, 5<) from the MOIs placed after the second stage of the 3-stage Harvard concentrator during each exposure period. The most distinct difference between the size distributions observed at the two sites was that the mass of particles <0.18 μm measured in Detroit were 3.6 times the mass measured in Grand Rapids and the mass of particles in the size range 0.6 > 0.18 μm were 1.9 times higher in Detroit than those measured in Grand Rapids. As a result, these two lower fractions accounted for 12.5% of the particle mass in Detroit compared to 6% in Grand Rapids.

**Table 1:** Size distribution of CAPs during 8-h exposure periods (MOIs measurements)

**Particle size (μm)**	**Detroit (%)**	**Grand Rapids (%)**
2. < 5	8.3	3.0
1.0 < 2.5	19.1	21.2
0.6 < 1.0	58.4	69.4
0.18 < 0.6	10.9	5.6
< 0.18	1.6	0.4

#### *CAPs chemical composition*

The chemical composition of Detroit CAPs was dominated by sulfates (39%) and organic carbon (32%), whereas CAPs in Grand Rapids were dominated by organic carbon (Table 2). Elemental concentrations at the two sites also revealed distinct differences. The concentrations of La, Pb, V, and Se in Detroit were 1.8, 2.2, 3.5, and 2.3 times higher, respectively, than those in Grand Rapids. In contrast, the concentrations of Ba, Ca, Mn, and Cu in Grand Rapids were 3.6, 1.5, 2.2, and 3.6 times higher, respectively than those in Detroit.

**Table 2:** CAPs composition and ambient gaseous pollutants during 8-h exposure periods in Detroit and Grand Rapids

		**Detroit**	**Grand Rapids**
CAPs mass	μg/m^3^	542	519
Organic carbon	μg/m^3^	177	280
Elementary carbon	μg/m^3^	4	7
Sulfate	μg/m^3^	216	58
Nitrate	μg/m^3^	19	23
Ammonium	μg/m^3^	48	51
Mg	ng/mg^3^	580	770
Al	ng/mg^3^	762	608
Ca	ng/mg^3^	1557	2365
V	ng/mg^3^	22	6
Mn	ng/mg^3^	108	236
Fe	ng/mg^3^	2760	3410
Ni	ng/mg^3^	9	10
Cu	ng/mg^3^	64	228
Zn	ng/mg^3^	594	438
Se	ng/mg^3^	141	61
Rb	ng/mg^3^	3.4	4.2
Mo	ng/mg^3^	4.6	5.2
Cd	ng/mg^3^	2.3	1.9
Ba	ng/mg^3^	7.0	25.1
La	ng/mg^3^	2.0	1.1
Ce	ng/mg^3^	3.0	3.7
Pb	ng/mg^3^	107	49
Ambient gaseous pollutants			
CO	ppm	0.6	1.2
SO_2_	ppb	29	2
NO	ppb	9	14
NO_2_	ppb	22	15

#### *Sources of PM*_*2.5*_

**Figure 7:**
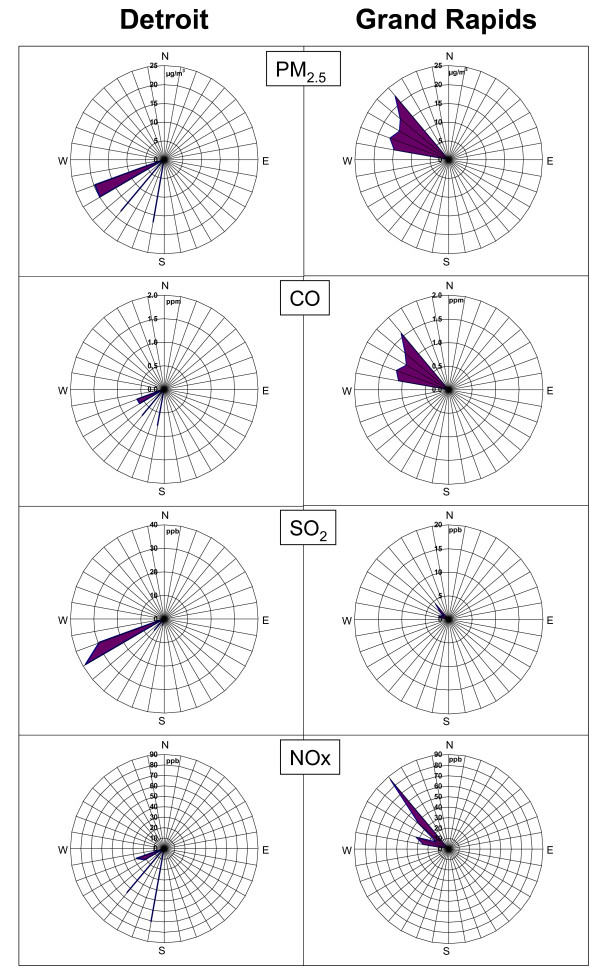
Pollutant concentrations versus wind direction during the 8-h exposure periods in Detroit and Grand Rapids.

Wind rose-plots (Figure 7) show distinct differences in time-averaged ambient PM_2.5_ and gaseous pollutant concentrations during exposures in Detroit versus Grand Rapids. During the Detroit exposure study period, high PM_2.5_ and SO_2_ concentrations were observed with west-southwesterly winds from the direction a number local industrial emissions source. In this direction ~ 250-270° is a large iron and steel facility and a major PM_2.5_ and SO_2_ emission source in Wayne County, emitting over 1000 tons of PM_2.5_ and over 200 tons of SO_2_ per year [19]. Located to the southwest of the exposure site are other large point sources including lime industries and well as oil refineries. In addition elevated PM_2.5_ and NO_X_ concentrations were observed with winds from ~ 220°, where the Detroit sewage sludge incinerator is located. The observations are consistent with the elevated elemental concentrations in CAPs of Pb (iron/steel manufacturing, and sewage sludge incinerator), La (refineries), V (refineries/sewage sludge incinerator), and Se (coal combustion, and sewage sludge incinerator).

Our previous results using Positive Matrix Factorization (PMF) receptor modeling on the samples collected during 2000–2003 studies [20] revealed that our Detroit exposure site often gets impacted by six major sources including coal/secondary sulfate aerosol, motor vehicle/urban road dust, municipal waste incinerators, oil combustion/refineries, sewage sludge incinerators, and iron/steel manufacturing. The PMF data indicate that iron/steel manufacturing, sewage sludge incinerators, and refineries together account for over 40% of the measured PM_2.5_ during the 8-h Detroit exposure in the present study. This further confirms that elevated metal concentrations in CAPs collected in Detroit are likely to be associated with local combustion sources as described above.

**Figure 8:**
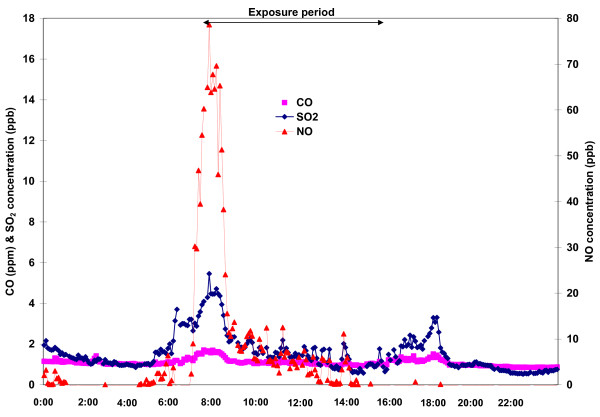
Time-series plot of CO, SO2 and NO concentrations in Grand Rapids before, during and after the 8-h inhalation exposure.

In comparison, in Grand Rapids the directionality plots of the time-averaged ambient PM_2.5_ and gaseous pollutant concentrations in Figure 8 show that high concentrations of PM_2.5_, CO and NOx were observed in northwesterly winds. This dominant wind direction placed our exposure site directly downwind from heavily trafficked highways in Grand Rapids. As described, this study location was within 60 m of one of the major roads in the city of Grand Rapids, and in particular the highest CO, NOx, and SO_2_ levels were observed during the morning rush hour (6:00–9:00 am, Figure8). Furthermore the organic carbon mass fraction of the PM_2.5_ was in excess of 50%, and was associated with elevated concentrations of Ba, Ca, Mn and Cu, which have been associated with brake wear and urban road dust [28,29]. Although we had insufficient sample size (n < 100) to conduct PMF analysis, these analyses suggest that gasoline- and diesel-powered vehicles were the dominant sources of ambient PM_2.5_ during our exposure in Grand Rapids.

## Discussion

Our previous results from CAPs exposure studies in Detroit suggest that exacerbation of allergic airway inflammation is associated with PM_2.5_ primarily associated with sewage sludge incineration, refineries, and secondary sulfate/coal-combustion utilities rather than from diesel- or gasoline-powered motor vehicles [15,16]. In the present study we again found PM_2.5_ -induced enhancement of allergic airway responses at our Detroit exposure site, but in contrast we also document inhibitory results by inhalation of Grand Rapids PM_2.5_ despite using the same exposure and treatment protocols and similar CAP mass concentrations (519 *μ*g/m^3^ vs 542 *μ*g/m^3^; Grand Rapids and Detroit, respectively). Because these divergent responses are independent of PM_2.5_ mass, then specific physicochemical attributes of the exposure aerosols must be driving the inhibition (Grand Rapids) and enhancement (Detroit) of allergic airway responses.

During the 8-h exposure period in Detroit southerly winds, associated with a high-pressure system centered over the Ohio River Valley, brought humid air masses and elevated levels of the transported or secondary particles dominated by sulfates to the exposure site. In addition to secondary/transported sulfate, increased concentrations of anthropogenic metals including Pb, V, and Se, suggest that the site was also impacted by emissions from the local industrial sources that we have identified southwest of the exposure study location. The potential combination of sulfates and metals to exacerbate allergic responses is consistent with results from a comparison study of PM_2.5_ collected from two German communities [30], which showed that PM samples with higher sulfates, Pb and other metals were more potent airway inflammagens in both allergic and normal mice. In another comparison study of PM_2.5_ collected from several European cities, the potency of PM to elicit immune response in mice was associated with V, Ni, and SO_4_ content of particles [31].

In contrast to the Detroit exposure, the dominant wind direction during the 8-h exposure period in Grand Rapids was northerly, bringing relatively clean air since there are few major emission sources in that direction. Temporal variations of NOx, CO and SO_2_ concentrations indicated a strong impact from the traffic in the morning. Therefore our observations for inhibition of eosinophil and mucus responses by Grand Rapids CAPs runs counter to epidemiological findings for traffic-associated asthma symptoms and hospitals visits [32-34]. These reported associations were specific for children, and asthma diagnoses were derived from pulmonary function endpoints and not from inflammatory cell infiltration and mucus production that we describe in rats. Results from animal models of allergic airways disease have reported both positive and negative correlations of airway inflammation (i.e., BALF cellularity) with altered airway function [35,36]. Another key difference is that our study addressed the effects of a single exposure to PM during the initial antigen challenge compared to chronic exposures of children to the daily variations of urban traffic, PM and allergens.

In past studies with multiple CAPs exposures (3–13 days) we have documented both enhancement, as well as no effects on allergic inflammatory responses in rats [14,17]. It was often challenging to decipher which exposure day had the most impact during those multi-day studies. The present comparison provides much clearer and distinct exposure profiles, as well as their opposing biological effects in animals.

Our results with Grand Rapids CAPs are reminiscent of our recent findings using the same Brown Norway –ovalbumin protocols where we used inhalation exposure to diesel engine exhaust (DEE) instead of CAPs. Inhalation of as little as 30 μg/m^3^ DEE also inhibited allergic airway inflammation and mucous cell metaplasia in asthmatic rats [15]. Components of diesel fuel emissions can induce Phase II enzymes in B-lymphocytes and inhibit IgE production [37]. We detected a decrease in OVA-specific IgE in serum of allergic rats exposed to Grand Rapids CAPs compared to allergic rats exposed to filtered air. These data are not shown because we did not conduct a similar analysis in Detroit to make a meaningful comparison. Several examples in airway cell culture systems suggest that PM_2.5_ -mediated depression of immune and inflammatory responses may be associated with oxidant capacity and toll-like receptor (TLR) activation [38-40]. These observations include inhibition of cytokine release or mediator production from airway epithelium or mononuclear inflammatory cells. Endotoxin from gram-negative bacteria, a TLR4 activator, can inhibit allergic inflammation during allergen challenge in ovalbumin-rat protocols [41]. We did not analyze PM samples for endotoxins in either Detroit or Grand Rapids, so it is possible that biogenic substances such as endotoxins may be present in PM where they contribute to inhibition of allergic responses.

Another notable difference between the exposures was that smaller size fractions of PM_2.5_ (<PM_0.6_) and ultrafine fraction (<PM_0.18_) was more than twice as high in Detroit CAPs compared to Grand Rapids. This finding suggests that impacts from local Detroit combustion sources were greater than from Grand Rapids. While the increased ultrafine concentrations could not be directly associated with the observed health effects in the present study, other studies have shown the size fraction may play an important role in understanding the PM health effects. For example, our recent study in Los Angeles suggests that ultrafine PM (100 μg/m^3^) can exacerbate allergic airway responses with repeated exposures before and during allergen challenge [42].

## Conclusions

Our results using two PM_2.5_ exposures of nearly identical mass concentrations but with distinct physicochemical constituents produced divergent results in experimental asthma outcomes. The clearest differences between these real-world PM_2.5_ exposures were high sulfate and metal concentrations (Detroit) which enhanced allergic airway responses, and high organic carbon (Grand Rapids) which led to inhibition of these responses. Inhibition by PM_2.5_ is associated with trace elements that are consistently linked to motor vehicle emissions without other significant identifiable sources of PM_2.5_. The inhibition of allergic inflammation by PM_2.5_ may be mediated as immune depression of airway macrophages and epithelium to appropriately respond to allergic and inflammatory stimuli. These results suggest that chemical components and size distributions of urban PM_2.5_ are more closely related than mass concentration to airway responses in asthmatics, and that the dose parameter of particle mass may not be adequate to evaluate adverse health effects that are associated with PM exposure. Together these data both enlighten and present interesting hypotheses for epidemiological associations of PM-associated asthma morbidity in urban settings.

## Abbreviations

PM: Particulate matter; PM_2.5_: Particulate matter in the size fraction ≤ 2.5 μm; CAPs: Concentrated air particles; OVA: Ovalbumin; BALF: Bronchoalveolar lavage fluid; IM: Intraepithelial mucosubstances; IN: Intranasal; DTM: Dry test meter; MOIs: Micro-orifice impactors; O_3_: Ozone; SO_2_: Sulfur dioxide; NOx: Nitrogen oxides; CO: Carbon monoxide; PMF: Positive matrix factorization; DEE: Diesel engine exhaust; TLR: Toll-like receptor.

## Competing interests

The authors declare that they have no competing interests.

## Authors' contributions

JW and JH designed and conducted the animal study. MM and JK planned and executed the exposure assessment and air quality monitoring. JW and MM took the leads on drafting the *in vivo *study results and the exposure assessment results in this manuscript, respectively. All authors reviewed the results of this manuscript, and JW, JH and MM approved the final manuscript.

GJK passed away prior to reviewing the final version.
